# Non-destructive ZooMS identification reveals strategic bone tool raw material selection by Neandertals

**DOI:** 10.1038/s41598-020-64358-w

**Published:** 2020-05-08

**Authors:** Naomi L. Martisius, Frido Welker, Tamara Dogandžić, Mark N. Grote, William Rendu, Virginie Sinet-Mathiot, Arndt Wilcke, Shannon J. P. McPherron, Marie Soressi, Teresa E. Steele

**Affiliations:** 1 Department of Anthropology, University of California, Davis, Davis, CA 95616-8522 USA; 2 Evolutionary Genomics Section, Globe Institute, University of Copenhagen, Øster Farimagsgade 5, 1353 Copenhagen, Denmark; 3 Department of Human Evolution, Max Planck Institute for Evolutionary Anthropology, Deutscher Platz 6, D-04103 Leipzig, Germany; 4 Department of Anthropology, University of Pennsylvania, 3260 South Street, Philadelphia, PA 19104-6398 USA; 5 Centre National de la Recherche Scientifique, MCC, Préhistoire à l’Actuel, Cultures, Environnement, Anthropologie, UMR5199, Université de Bordeaux, FR-33615 Pessac, France; 6 Fraunhofer Institute for Cell Therapy and Immunology, Perlickstraße 1, D-04013 Leipzig, Germany; 7 Faculty of Archaeology, Leiden University, 2300 RA Leiden, The Netherlands

**Keywords:** Mass spectrometry, Archaeology, Archaeology

## Abstract

Five nearly identical fragments of specialized bone tools, interpreted as *lissoirs* (French for “smoothers”), have been found at two Middle Paleolithic sites in southwest France. The finds span three separate archaeological deposits, suggesting continuity in the behavior of late Neandertals. Using standard morphological assessments, we determined that the *lissoirs* were produced on ribs of medium-sized ungulates. However, since these bones are highly fragmented and anthropogenically modified, species determinations were challenging. Also, conservative curation policy recommends minimizing destructive sampling of rare, fragile, or small artifacts for molecular identification methods. To better understand raw material selection for these five *lissoirs*, we reassess their taxonomy using a non-destructive ZooMS methodology based on triboelectric capture of collagen. We sampled four storage containers and obtained identifiable MALDI-TOF MS collagen fingerprints, all indicative of the same taxonomic clade, which includes aurochs and bison (*Bos* sp. and *Bison* sp.). The fifth specimen, which was stored in a plastic bag, provided no useful MALDI-TOF MS spectra. We show that the choice of large bovid ribs in an archaeological layer dominated by reindeer (*Rangifer tarandus*) demonstrates strategic selection by these Neandertals. Furthermore, our results highlight the value of a promising technique for the non-destructive analysis of bone artifacts.

## Introduction

It is now accepted that some Neandertals produced bone tools^1–3^. These include the discovery of four specialized bone tools from a total of three layers at two late Middle Paleolithic sites in southwest France, Pech-de-l’Azé I (Pech I) and Abri Peyrony^4^. Subsequently, a fifth example was found at Abri Peyrony^5^. We interpreted these fragments of shaped and worn animal ribs as *lissoirs*, a term meaning “smoothers” in the French bone tool typology^6^. Analyses suggest that these tools preserve traces consistent with abrasive pressure on animal skin^4,5^. The use of these tools during the hide working process reflects a complex technical system where animal ribs were exploited for their specific raw material properties^7^. Based on standard zooarchaeological assessments, their width and thickness suggest that they might have been produced from ribs of medium-sized ungulates (hooved animals such as red deer (*Cervus elaphus*) and reindeer (*Rangifer tarandus*)). However, taxonomic assignments of bone tools are difficult as their production and use often involves the deliberate reshaping of external surfaces, removing diagnostic features. Modified ribs present additional challenges due to shape and size variation across the ribcage. Though standard zooarchaeological analyses are needed to assess certain aspects of raw material selection (e.g., skeletal element), Zooarchaeology by Mass Spectrometry (ZooMS), a mass-spectrometric method for identifying tissues rich in collagen type I (COL1) to family or genus level^8^, provides a means to evaluate which animals were selected. Here, we assess the taxonomy of the five *lissoirs* using a non-destructive ZooMS approach^9^.

ZooMS has been useful for identifying ancient animal remains when fragmentary bones present a challenge for more traditional methods. The method has been used to identify fragmented animal and human remains from Paleolithic sites^10–17^, as well as cultural artifacts from various time periods, including bone tools^9,18–24^ and parchment^25^. Such objects frequently have been significantly altered from their original form making taxonomic identifications based on morphology nearly impossible. At the same time, conventional ZooMS extraction procedures involve drilling or cutting a bone sample (<20 mg), thereby altering often unique and fragile artifacts (Supplementary Fig. S1). Recently, a non-destructive approach based on the triboelectric effect occurring between collagen and plastic surfaces has been developed to sample parchment for ZooMS analysis^25^. The concept behind this approach has been applied to plastic storage bags containing bone artifacts as well^9^, as the use of erasers carries the risk of modifying bone surfaces through abrasion. Here, we show that collagen molecules adhering to the plastic surfaces allow us to infer species selection of Middle Paleolithic *lissoirs* made by Neandertals, which then permits the consideration of competing hypotheses about the selection of ribs as the raw material for making *lissoirs*.

## The Middle Paleolithic *Lissoirs*

*Lissoirs* are specialized bone tools likely shaped for the purpose of working animal skins. Although these tools have been defined in various ways, they are typically characterized as elongated rib fragments with rounded distal ends that often exhibit polish^26–31^. The shape of *lissoirs* is largely reflective of the rib form. Minimal shaping is needed to obtain a rounded and usable active end, and the rest of the rib need not be shaped at all. Additionally, ribs are mechanically suitable for technological purposes. A particular advantage of the structure of rib bones is the double layer of cortical bone sandwiching a layer of cancellous bone^32^. This composite structure provides stiffness and strength during bending, meaning that the bone can withstand substantial force but will return to its original shape once force is no longer applied^32,33^. The versatile properties of ribs may have been a factor in their selection as a raw material for tool use.

The five Middle Paleolithic *lissoirs* in this study come from three separate archaeological layers at two sites in southwest France: Pech I and Abri Peyrony. The two sites are on different tributaries of the Dordogne river approximately 35 km from one another^4^. Pech I is one of four rock-shelter sites within the Pech-de-l’Azé complex^34–36^. In the original 1909 excavation, the remains of a Neandertal child were discovered^37,38^. The site was then re-excavated several times^34,39–41^, and the most recent excavations resumed in 2004^35^. There are three main layers, and the *lissoir*, G8-1417, is attributed to Layer 4 located directly above the bedrock. Layer 4 dates to 51.4 ± 2.0 ka through single-grain optically stimulated luminescence dating^4,42^, which is consistent with other dating methods used on the site^38^. Layer 4 consists of a clayey sand matrix that derives from the underlying endokarstic sediments^35,43^. The layer is minimally disturbed as indicated by *in situ* anatomical bone connections and broken artifacts, and there exists little evidence of trampling traces (<1%) on the bones. The lithic assemblage consists of artifacts typical of the Mousterian of Acheulian Tradition including backed knives and cordiform handaxes.

Abri Peyrony is located against a cliff overlooking the Couze valley. It was initially excavated by Peyrony^44^ as a part of the Combe Capelle complex of sites. In 1990, Dibble and Lenoir^45^ conducted a limited test excavation at the site, and the most recent excavations were conducted between 2009 and 2012^4^. The site is divided into two sectors corresponding to an upper and lower terrace. The majority of archaeological material comes from the lower terrace. The four *lissoirs* derive from two lower terrace (L-) layers, L-3A and L-3B, with the latter unit situated on the bedrock. Three of the bone artifacts were recovered from Layer L-3B (AP-4493, AP-7839, AP-10818), while one was found in Layer L-3A (AP-4209). Radiocarbon age determinations on the two layers produced no statistical differences, and together the Layer L-3 ages range from 47,710–41,130 cal B.P. Limestone fragments coming from the cliff are found throughout the layers and calcium carbonate from groundwater cemented the overlying layer soon after deposition. The cementation process likely prevented the downslope movement of the layer and perhaps many other post-depositional disturbances^4^. Additional evidence that the layer represents a primary depositional context and that it is minimally disturbed comes from an intact combustion feature and both bone and lithic refits^46^. Abri Peyrony is known for its Mousterian of Acheulian Tradition stone tools^44^. In our renewed excavations we found more variability than has been previously described. The lithic assemblage from L-3A includes backed knives, cordiform handaxes, and handaxe thinning flakes, all of which are consistent with a Mousterian of Acheulian Tradition attribution. However, while the L-3B lithics show some overall similarities to L-3A, there are no backed knives and no clear evidence of handaxe production typical of the Mousterian of Acheulian Tradition. Rather, this layer is characterized by the discoidal flaking method and an increase in denticulate and notched tools compared to the L-3A assemblage.

**Figure 1:**
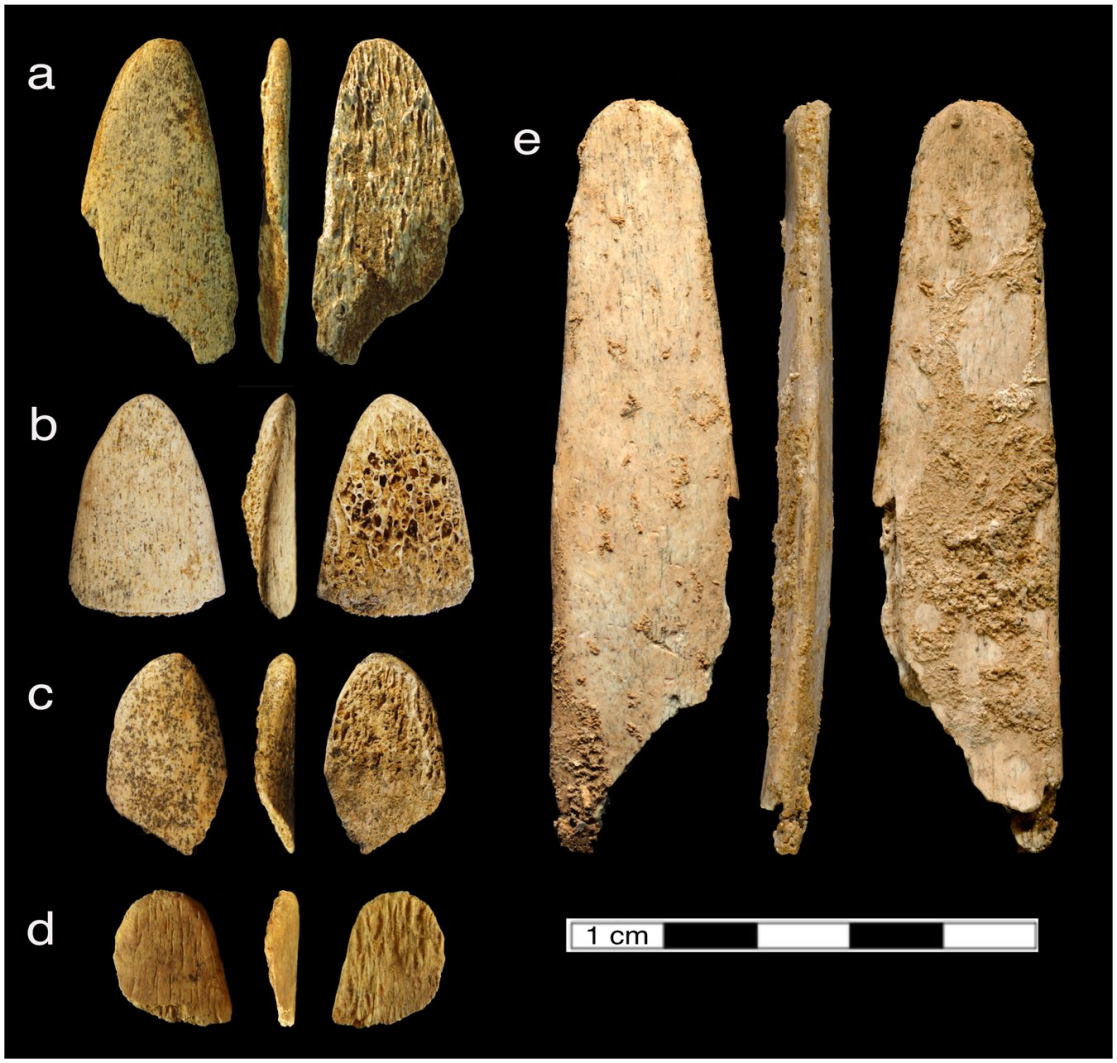
Photographs of the (**a**) Pech-de-l’Azé I (PA I) and (**b-e**) Abri Peyrony (AP) *lissoirs*. (**a**) PA I G8-1417. (**b**) AP-4209. (**c**) AP-4493. (**d**) AP-10818, newly published here. (**e**) AP-7839. Adapted/modified from^4^.

Our previous study^4^ demonstrated that the Pech I *lissoir* and three Abri Peyrony *lissoirs* share characteristics such as a rounded, ogival distal end with a polished cortical surface that extends partially along the edges. An additional *lissoir* (AP-10818) found in the screened material from Layer L-3B at Abri Peyrony (Fig. 1d) exhibits characteristics most similar to AP-7839 (Fig. 1e). Parallel striations along artifact edges and surfaces are consistent with manufacturing traces by grinding against a coarse material like sandstone^4,5^. The artifacts preserve microscopic characteristics such as intrusive smoothing of the upper reliefs and furrows of the microtopography and various types of striations. These characteristics are consistent with use on a soft material such as animal skin^4,5^.

Though morphological assessments indicated that all of the Middle Paleolithic *lissoirs* were made from ungulate ribs, the fragmentation and modification of the artifacts made species determinations challenging. The application of ZooMS provides us with the ability to ascertain species selection by Neandertals for making *lissoirs*. Given that Middle Paleolithic *lissoirs* are rare, we consider another Neandertal bone tool as a baseline for Neandertal selectivity. Specifically, bone retouchers—bone fragments opportunistically utilized to shape flint artifacts—have been shown to originate from commonly hunted species, and their taxonomic frequencies are similar to the overall distribution of taxa in faunal assemblages^47–49^ (but see^50,51^). It is possible that Neandertals showed a similar lack of species selectivity when utilizing ribs—the raw material source for making *lissoirs*. Alternatively, Neandertals used the same skeletal element (rib) for making *lissoirs*, which provides a line of evidence for Neandertal selectivity. In addition, the highly standardized final shape and size of the five Pech I and Abri Peyrony *lissoirs* could imply that Neandertals selected the ribs from species of a certain size. Thicknesses of the *lissoirs* (Supplementary Table S1) could be consistent with large ungulate rib dimensions, which would make them stronger implements. If Neandertals consistently selected the ribs of the larger animals in their environment, this could provide evidence for strategic raw material selection. Given this, applying non-destructive ZooMS to the five Middle Paleolithic *lissoirs* provides an opportunity to test competing propositions about Neandertal selection of ribs for technological purposes. We consider the following hypotheses: Null (*H*_0_): Neandertals in southwest France selected ribs for *lissoirs* opportunistically. Alternative (*H*_*A*_): Neandertals in southwest France selected ribs of larger species for *lissoirs* strategically. We address these hypotheses using the results of the zooarchaeological and ZooMS analyses.

## Results

### The zooarchaeological assemblages

The Pech I Layer 4 fauna is well-preserved and primarily consists of red deer elements (51% NISP) with the second most abundant species represented by large bovids such as bison (*Bison priscus*, 18% NISP). Other ungulates including roe deer (*Capreolus capreolus*), reindeer, and horse (*Equus*) are represented in smaller frequencies (Table 1)^52^. Abri Peyrony bone preservation is good with minimal weathering. In Layer L-3A, medium-sized ungulates such as reindeer and red deer dominate the assemblage (63% NISP combined) with large bovids (aurochs, *Bos* sp./bison, *Bison* sp.) also well-represented (29% NISP). Roe deer are present in smaller proportions (Table 2). Conversely, L-3B is almost entirely dominated by medium ungulates likely to be reindeer (90% NISP) with large ungulates represented at about 9% (3% are specifically attributed to large bovids; Table 2). The large ungulates present in the region during this time period are large bovids and horses, but of these, only large bovids have been confidently identified at Abri Peyrony.

**Table 1:** Number of Identified Specimens (NISP) and percentage of each taxon represented in the assemblage identified through morphological assessments, in the *lissoir* bearing layer at Pech I.

Taxon	Common Names	Pech I 4	
		NISP	% NISP
*Vulpes vulpes*	fox	1	<1%
*Lepus* sp.	hare	2	<1%
*Capreolus capreolus*	roe deer	44	7% (3%)
*Cervus elaphus*	red deer	327	51% (13%)
*Rangifer tarandus*	reindeer	12	2%
*Cervus*/*Rangifer*	red deer/reindeer	12	2%
*Bison* sp./*Bos* sp.	bison/aurochs	114	18% (10%)
*Equus caballus*	horse	6	<1%
	small ungulate (ungulate size class 1)	1	<1%
	ungulate size class 1/2	2	<1% (1%)
	medium ungulate (ungulate size class 2)	59	9% (33%)
	ungulate size class 2/3	16	2% (15%)
	ungulate size class 2/4	15	2%
	large ungulate (ungulate size class 3/4)	29	5% (24%)
	avifauna	2	<1%
	**Total NISP**	**642 **	
	indeterminate	2010	
	**Total fragments**	**2652**	

The numbers of identified ribs of each taxon are displayed in brackets. Rib percentages are calculated from the total number of ribs in each layer and are displayed in parentheses.

**Table 2:** Number of Identified Specimens (NISP) and percentage of each taxon represented in the assemblage identified through morphological assessments, in the *lissoir* bearing layers at Abri Peyrony.

Taxon	Common Names	Abri Peyrony L-3A		Abri Peyrony L-3B	
		NISP	%NISP	NISP	%NISP
*Lepus* sp.	hare			3	<1%
*Capreolus capreolus*	roe deer	4	6%	1	<1%
*Cervus elaphus*	red deer	7	10%		
*Rangifer tarandus*	reindeer	12	18%	264*	49% (2%)
*Cervus*/*Rangifer*	red deer/reindeer	22	32% (40%)	219	40% (88%)
Cervid	cervid (antler tips)	1	1%	1	<1%
Cervid/Saiga	cervid/saiga	1*	1%		
*Bison* sp./*Bos* sp.	bison/aurochs	10*	15% (40%)	16	3% (7%)
	large ungulate	10	15% (20%)	35	6% (2%)
Rhinocerotid	rhinoceros			1	<1%
	medium carnivore			1	<1%
	large carnivore	1	1%		
	Total NISP	68		541	
	indeterminate	316		834	
	Total fragments	384		1375	

In addition to the *lissoirs*, three additional identifications indicated with * were made using ZooMS (Supplementary Table S4). The numbers of identified ribs of each taxon are displayed in brackets. Rib percentages are calculated from the total number of ribs in each layer and are displayed in parentheses.

Because fragmentary ribs are often difficult to identify to species, most ribs were identified to body size class. Generally, the taxonomic distribution of ribs follows the frequencies of the overall body size class distribution in the faunal assemblages (Supplementary Fig. S2). The layer where the rib taxonomic distribution differs the most from the overall faunal distribution is L-3A at Abri Peyrony. This is also the layer with the fewest identified specimens (*n* = 68), so the difference may be merely a stochastic feature of the small sample.

### Taxonomic identifications through ZooMS

In order to non-destructively sample the Middle Paleolithic *lissoirs*, objects that had extended contact with the artifacts were obtained for sampling. The four Abri Peyrony *lissoirs* were stored in separate plastic curation boxes, within which the *lissoirs* were suspended between two flexible polyurethane membranes (so-called membrane boxes) for different lengths of time (Table 3; Supplementary Fig. S3). Each box had not been used to store other specimens prior to the *lissoirs*. A membrane box was not used to curate the Pech I *lissoir*, so we sampled an acetate surface replica made for a use-wear analysis^4^, as well as a plastic bag that stored the *lissoir* for about five months (Table 3).

**Table 3:** Site, assemblage, and *lissoir* sampling information.

Site and Layer	Lithic technology	Dominant fauna taxa	Absolute dates	Specimen ID number	Sample type	Time span in contact with bone	ZooMS ID
Pech-de-l’Azé I 4	Bifacial handaxes, backed knives, laminar and some Levallois-like blanks (Mousterian of Acheulean Tradition)	Red deer and bison	51.4 ± 2.0 ka	PA I G8-1417	1) Acetate surface replica (with and without cutoff piece)	A few minutes	Unidentifiable
					2) Plastic bag	~Five months	Unidentifiable
Abri Peyrony L-3A	Bifacial handaxes, backed knives, Levallois (Mousterian of Acheulean Tradition)	Reindeer, red deer, and large bovids	47,710–41,130 cal B.P.	AP-4209	Membrane box	>Five years	*Bos* sp./*Bison* sp.
Abri Peyrony L-3B	Discoidal and Levallois methods, denticulate tools	Reindeer (note the large bovid minor component)	47,710–41,130 cal B.P.	AP-4493	Membrane box	>Five years	*Bos* sp./*Bison* sp.
				AP-7839	Membrane box	>Five years	*Bos* sp./*Bison* sp.
				AP-10818	Membrane box	~Two months	*Bos* sp./*Bison* sp.

Site and assemblage information including lithic technology, dominant fauna taxa, and absolute dates.

*Lissoir* specimen information including sampled membrane box, plastic bag, or acetate surface replica used in this study; length of time sample was in contact with each *lissoir*; and ZooMS taxonomic identity.

All membrane boxes containing the Abri Peyrony *lissoirs* produced the same taxonomic identification of *Bos* sp./*Bison* sp., while the Pech I acetate replica and plastic bag provided no taxonomic indicators due to multiple empty spectra (Table 3; Supplementary Table S2). We exclude an alternative attribution to musk ox (*Ovibos moschatus*) for AP-7839 and AP-10818 as this species is not known from the region and time period (Supplementary Table S3)^53^.

**Figure 2:**
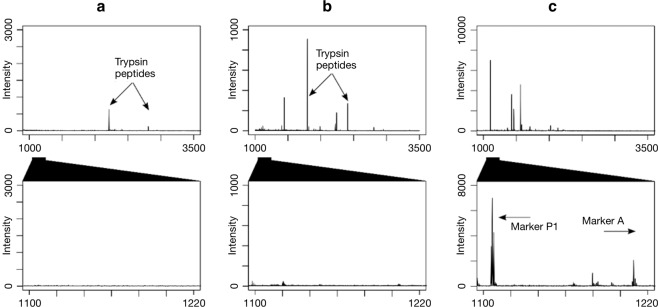
Examples of non-destructive MALDI-TOF MS spectra. (**a**) Membrane extraction blank. (**b**) Washing control #2 from Les Cottés. This spectrum represents one example of eight washing and sedimentary control samples processed. (**c**) *Lissoir* (AP-4209) from Abri Peyrony. The top row displays the complete spectrum in the m/z range 1000 – 3500. The bottom row displays a close-up view of the m/z range around peptide marker P1 (1105 m/z) and A (1208 m/z for *Bos* sp./*Bison* sp.). Note the difference in peptide intensity between the controls and the *lissoir*.

Of note is the general absence of heavy m/z peptides in the MALDI-TOF MS spectra of the Abri Peyrony *lissoirs* (>2500 m/z; Fig. 2c; Supplementary Table S2). This could potentially be due to the triboelectric effect only allowing capture and transfer of low-molecular weight peptides, which has previously been observed for non-destructive extractions^9^. Such biased peptide preservation or peptide recovery could only generalize the achieved taxonomic identities to less-specific ones and cannot direct the taxonomic identity to any preferential class of mammals. For example, the plastic bag-based identifications of control samples G8-704 (Cervid/Saiga/Roe deer/*Capra* sp.), and AP-6953 and AP-5661 (both Bovidae/Cervidae) could not be specified further due to an absence of peptide markers G, and D to G, respectively (Supplementary Table S4). However, even these identifications provide some level of taxonomic information that is consistent with the morphological analysis.

### Contamination

Seven lines of evidence suggest that contamination did not contribute to our ZooMS taxonomic identifications. First, an extraction blank processed alongside the Middle Paleolithic *lissoirs* is empty of COL1. Second, an identical extraction performed on an unused membrane box contained no COL1 peptides either (Fig. 2a). Combined, these two lines of evidence exclude laboratory or membrane box contamination as an explanation of our results.

Third, we analyzed the water from washing bone artifacts as well as archaeological sediments to control for (cross-)contamination between bone specimens. Our washing and sediment control samples contain no COL1 peptides, in particular featuring an absence of *Bos* sp./*Bison* sp. or human-specific peptide marker masses (Fig. 2b). This indicates that our *lissoir* data are unlikely to derive from neither the initial, on-site washing of bone artifacts in shared wash water (all but one of the *lissoirs* were discovered after water-screening bulk sediments), nor from archaeological sediments.

Fourth, our peptide matching approach in mMass provides peptide marker matches to *Bos* sp./*Bison* sp. COL1 only, and not to any other taxa present in the peptide marker database^13^. This includes other ungulates, whose skin could have been worked by the *lissoirs*, or human COL1, which could be present on the *lissoirs* either through Neandertal handling or by modern contamination. Instead, the observed COL1 peptide marker series is *Bos* sp./*Bison* sp. specific (Fig. 2c, Supplementary Table S2).

Fifth, we only confidently match peptides deriving from COL1, porcine trypsin, and human keratins (keratin 1 and keratin 10). The absence of multiple confident matches against peptides deriving from *Bos* albumin, *Bos* hemoglobins (*HBA* and *HBB*), and *Bos*-specific keratins indicates that no skin proteins surviving from *Bos* hide working are present in the MALDI spectra. Based on sequence homology, we infer that it is therefore also highly unlikely that skin proteins deriving from other ungulates are present in the spectra. The absence of skin proteins is potentially due to the enhanced preservation of mineral-bound proteins involved in biomineralization (such as collagens)^54,55^. In contrast, proteins not involved in mineral-binding are preferentially lost across archaeological time scales. This would include usage-derived or handling-derived proteins, which might be confidently identified using more sensitive nanoLC-MS/MS. Their observation would not, however, change the taxonomic identity of the bone artifact studied.

Sixth, previous studies utilizing the triboelectric effect for collagen peptide mass fingerprinting on both parchment^25^ and bone tools^9^ were able to verify their non-destructive identifications with destructive proteomic and genomic analyses. We could not verify our ZooMS taxonomic identities of the *lissoirs* by directly sampling them for ancient DNA or collagen analysis, as this would irreversibly damage these artifacts (Supplementary Fig. S1). Instead, we sampled 24 bone specimens from Abri Peyrony and 16 bone specimens from Pech I, the majority of which had known taxonomic identities based on bone morphology (Supplementary Table S4). For Abri Peyrony, we performed destructive ZooMS analysis, and extractions on membrane boxes and plastic bags that contained these bone specimens. For Pech I, we only tested bag-based triboelectric extraction. When destructive analysis was successful, its taxonomic assignment was most often in agreement with the morphological taxonomic assignment (Supplementary Table S4). However, two specimens (AP-4183 and AP-5661) morphologically identified as *Bison* sp./*Bos* sp. consistently provided ZooMS identities of differing taxa (cervid/saiga and reindeer), which indicates that the original morphological identifications were incorrect. ZooMS applied to plastic bags and membrane boxes that stored the bone specimens were less successful. However, when they did provide results, they were in agreement with the morphological and destructive ZooMS identities (Supplementary Table S4). The exception is one specimen, which was identified morphologically as reindeer (AP-3597), whose plastic bag extraction resulted in assignment to Ursidae. The destructive extraction performed on the specimen informs us that, in fact, it contains no collagen type I. It is therefore likely that the collagen profile obtained from the plastic bag derives from contamination prior to laboratory analysis (a deamidation value of 1.0 supports this interpretation; see Supplementary Fig. S4), which could have happened in case the plastic bag was used to (temporarily) store another bone specimen prior to holding specimen AP-3597.

Seventh, our deamidation calculations for the control specimens of Abri Peyrony, extracted by destructive and non-destructive methods, indicates the presence of degraded collagen (Supplementary Fig. S4). Values of the destructive (ranging from 0.29 to 0.53) and non-destructive extraction approaches (0.23 to 0.55) are comparable. Due to limited collagen recovery for both non-destructive methods, there are few comparative data points available. This extends to the *lissoirs*, for which we can only reliably calculate deamidation of the P1105 peptide for one *lissoir*. The value obtained for this specimen (AP-7389, 0.45) also indicates the presence of degraded collagen type I and falls within the range observed for control bone specimens from the same site.

In summary, our tests on faunal bone specimens from both Abri Peyrony and Pech I indicate that plastic-bag and membrane-box approaches provide reliable taxonomic identifications. This extends to the deamidation values obtained for the non-destructive methods, as they fall in the same range. At the same time, the limited comparative data at hand suggests that success rates using both non-destructive approaches are low and variable. Further work should clarify the circumstances (for example sedimentary, chronological, or plastic surface composition) under which either approach is preferable, and whether it is possible to improve success rates by changing extraction chemistries.

These seven lines of evidence rule out the possibility that the ZooMS profiles from the membrane boxes that held the Middle Paleolithic *lissoirs* originate from bone tool use in the past or from modern contamination, either in the field or in the laboratory. Therefore, any collagen identified from the membrane boxes must come from the *lissoirs* themselves.

### Relative support for competing hypotheses

Availability, suitability of skeletal element shape, and quality of the bone element and/or species^56^ are important characteristics when selecting a raw material source to make bone tools. Whether Neandertals took any of these factors into account when selecting, making, and using bone tools is a matter that needs further consideration. The ability to discriminate species through the application of ZooMS provides an opportunity to test competing propositions about Neandertal selection of ribs for functional purposes (*H*_0_: opportunism vs. *H*_*A*_: selection of larger ribs). We employ Bayesian reasoning to quantify the relative support for competing hypotheses in light of the observed faunal distributions and ZooMS results^57,58^. Our statistical framework incorporates both a *strong* alternative *H*_*As*_, implying that Neandertals had relatively fixed preferences for ribs of larger-bodied ungulates, and a *weak* alternative *H*_*Aw*_, implying less rigid preferences (Supplementary Note).

**Figure 3:**
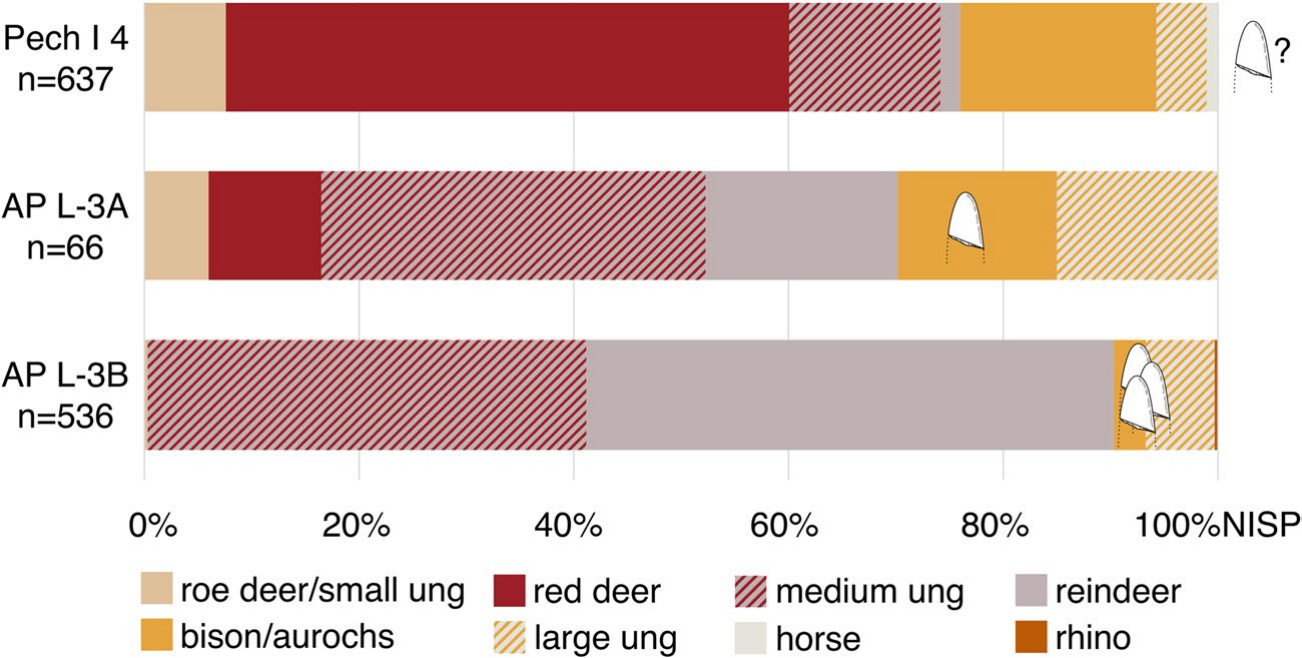
Ungulate species composition by the number of identified specimens (NISP) of the layers that preserved the *lissoirs* at Pech I and Abri Peyrony (AP); *lissoirs* indicated by their taxonomic identity as observed through ZooMS. Specimens not identified to species, such as medium and large ungulates (ung), are depicted with diagonal stripes between their most likely attributed species. For simplicity, categories “red deer/reindeer”, “cervid/saiga”, and “medium ungulate” in Tables 1 and 2 are combined into “medium ungulate” here. *Lissoir* depictions courtesy of Anna E. Goldfield.

Only a single *lissoir* was recovered from Layer L-3A, in which large bovids are well-represented (Table 2). This limited evidence does not discriminate either hypothesis sharply, although H_0_ is better supported, with *P*(*E* | *H*_0_) = 0.6 compared to *P*(*E* | *H*_*A*_) = 0.125 (with a single observation, the likelihood under H_A_ is the same for both the strong and weak versions). The posterior odds of the alternative hypothesis for Layer L-3A are subsequently *H*_*A*_:*H*_0_ = 0.2 (Supplementary Note). In contrast, three *lissoirs* were recovered from Layer L-3B; this layer is dominated by reindeer with a much smaller representation of large bovids (Table 2; Fig. 3). The likelihood under H_0_ [*P*(*E* | *H*_0_) = 0.0009] reflects the unlikely event that three randomly selected ribs from this assemblage would be from large bovids (Supplementary Table S5). In contrast, the likelihoods under the strong (*H*_*As*_) and weak (*H*_*Aw*_) alternatives are *P*(*E* | *H*_*As*_) = 0.0028 and *P*(*E* | *H*_*Aw*_) = 0.0037, respectively. The posterior odds are subsequently *H*_*As*_:*H*_0_ = 3.2 and *H*_*Aw*_:*H*_0_ = 4.2, values greater than 1 indicating that in either case the alternative is better supported (Supplementary Note). Therefore, the *lissoir* sample in Layer L-3B is more consistent with some degree of preference for ribs of larger-bodied taxa, rather than with opportunistic selection dependent only on the relative frequencies of prey taxa.

## Discussion

The presence of four *lissoirs* in two separate layers (L-3A and L-3B) with distinct faunal distributions and lithic technologies (Fig. 3; Table 3) indicates that the *lissoir* technology persisted at Abri Peyrony across at least two time periods and environments. Even though multiple components of the assemblage changed across the two layers, the raw material used to make the *lissoirs* remained constant. The presence of three *lissoirs* in the reindeer-dominated layer supports the assertion that Neandertals were purposefully selecting large bovid ribs regardless of encounter frequencies during hunting. While there is some evidence demonstrating that Neandertals had access to large bovids during this period, Neandertals hunted them less frequently compared to reindeer (Fig. 3; Table 2).

Studies on modern reindeer and bison indicate that they have differing ecological needs but can adapt to similar environmental conditions^59^. Nonetheless, a dominant reindeer presence in archeological deposits, such as in Layer L-3B, indicates a colder overall environment^60–63^. In addition, optimal foraging theory suggests that hunters would likely pursue the highest ranked resource until depleted^64,65^. Based on body mass, large bovids were higher ranked and would be hunted whenever found on the landscape, but reindeer overabundance in Layer L-3B indicates that they were likely the most frequently encountered prey. It is therefore of interest to note that Neandertals ignored reindeer ribs and instead utilized the ribs of large bovids to make *lissoirs*. Though the sample size is small, the pattern at Abri Peyrony suggests intentional selection of ribs from the larger-bodied bovids. Neandertals likely collected ribs on the occasions that large bovids were encountered during hunting, but at this time, we cannot rule out curation of *lissoirs* made in different environmental settings.

The composite nature of bone, composed of an organic component (mostly collagen type I) and a mineral component (mainly calcium phosphate)^32^, make it extremely useful as a raw material, though these structural properties may differ depending on skeletal element^66,67^. The bone material strength of any skeletal element is relatively similar across most terrestrial taxa^32^. Size is the main inter-species difference in bone properties of a given element. Larger bones, logically, will be stronger and stiffer^67,68^. Neandertals at Abri Peyrony likely desired specific raw material properties when choosing the ribs of large bovids over smaller animals such as reindeer. The clear preference for the stronger rib could indicate that the tools were typically subjected to a substantial amount of pressure or were used repeatedly. The thinner cortical bone of a reindeer rib would likely fracture more quickly if used in the same way.

Given that the time period represented by Layer L-3B was likely fairly cold, it is possible that Neandertals sought out animal skins and prepared them more frequently. Even though Neandertal physiology is proposed to be cold adapted^69,70^, it has been suggested that Neandertals would have needed clothing and footwear, especially during the colder time periods^71–73^. It is reasonable to assume that Neandertals may have been working and preparing additional animal hides during cold climate phases, therefore requiring the repeated use of hide working tools such as *lissoirs*. The production and use of these tools are likely embedded in a broader technological strategy where Neandertals hunted large bovids for subsistence purposes and then selected their ribs and possibly their hides during the process.

There are other possible examples of Middle Paleolithic *lissoirs* made on large ribs. In 1907, a probable complete *lissoir* was found in the upper layers of La Quina^74^. The distal end morphology of the La Quina *lissoir* is nearly identical to those from Pech I and Abri Peyrony (Supplementary Fig. S5). Through morphological assessment, which included the articular head of the rib, this bone tool was identified as originating from the 10th left rib of a large bovid^74^. Because this artifact was complete, the morphological identification is more reliable than identifications made on fragmented artifacts. The unclear placement of the *lissoir* in the upper layers at La Quina makes discussing rib selection for functional purposes at La Quina difficult. Nonetheless, this artifact provides us with an independent line of evidence demonstrating that Neandertals made *lissoirs* from large bovid ribs within two regions in France.

Pech I, Abri Peyrony, and La Quina represent the best documented examples of *lissoirs* in Middle Paleolithic contexts, but two other possible examples should be mentioned. A potential *lissoir* from Grosse Grotte in southwestern Germany has been described as a mammoth (*Mammuthus primigenius*) rib fragment showing “typical modifications” for preparing animal skins^75^. Another possible *lissoir* from L’Abri de Canalette in southern France^76^ was described as coming from a red deer rib. Further analysis including ZooMS should help confirm taxonomic assessments, as based on our ZooMS results, we caution against the use of morphology alone in the taxonomic identification of highly modified bone artifacts. Once artifact and species attributes are verified, a comparison to faunal distributions from those sites would help assess Neandertal selection in other regions. It is possible that rib selection was not species-specific but reflects a preference for the strongest ribs of some of the largest animals in the environment. In addition, future applications could include addressing whether *Homo sapiens* in other time periods showed a similar preference. A thorough assessment employing ZooMS could provide insight into the human selection of raw materials and how their decision-making compares to the selection processes by Neandertals.

## Conclusion

The ZooMS methodology employed here provides a promising avenue for the entirely non-destructive sampling of bone artifacts including those that may be fragile, rare, or small. We demonstrate that this approach works on Middle Paleolithic artifacts stored in plastic containers for various lengths of time, allowing taxonomic identification of bone artifacts otherwise inaccessible for destructive molecular analysis. Our approach produced identifiable MALDI-TOF MS collagen fingerprints for all of the samples that held the Abri Peyrony *lissoirs*. Significantly, all four produced the same taxonomic identification (*Bos* sp*./Bison* sp.). We also caution, however, that such non-destructive approaches should only be applied to plastic bags or membrane boxes not previously used to store other (organic) artifacts. In addition, at the moment the success rates of such non-destructive approaches appear variable and inconsistent, and provide only partial MALDI-TOF MS spectra biased towards low-molecular weight peptides.

Our results demonstrate that Neandertals at Abri Peyrony utilized large bovid ribs to make *lissoirs* across two archaeological deposits with varying lithic technologies and faunal distributions. Significantly, the majority of the bone tools were preserved in Layer L-3B, a deposit dominated by reindeer remains. The incongruent taxonomic identifications of the *lissoirs* compared to the faunal composition of the layer suggests a preference for large bovid ribs as raw material for functional purposes. Large bovids ribs are stronger and more resilient to the application of bending force, a property that was likely valued during use of this technology. The selection of large bovid ribs for manufacturing *lissoirs* was likely repeated at least one other time at La Quina, which could indicate a more widespread behavioral pattern. Additional samples from secure archaeological contexts will help clarify the extent to which the broader Neandertal population was selective in the production of bone tools.

## Methods

### *ZooMS* analysis

Conventional ZooMS analysis requires drilling or cutting a small bone sample (<20 mg) from a larger specimen. Such procedures have the potential to significantly alter the object’s physical appearance. Instead, we utilized and modified a recently introduced approach of entirely non-destructive collagen extraction through triboelectric effects between plastic surfaces and bone collagen^9^. For the Abri Peyrony *lissoirs*, we sampled the four membrane boxes that stored the bone artifacts for various lengths of time. For the Pech I *lissoir*, we studied both an acetate surface replica as well as a plastic storage bag (Table 3).

Each membrane box that contained an Abri Peyrony *lissoir* was opened and the artifact removed. 100 μl ammonium-bicarbonate (pH = 8, 0.05 M), heated to 65 °C, was dragged across both membrane surfaces using a standard pipette. While dispersing the ammonium-bicarbonate buffer across the membrane, a continuous cycle of aspiration and release of the buffer was used to maximize the recovery of any microparticles or collagen molecules adhering to the membrane surface. The extraction procedure is thereby based on triboelectric effects between plastic polymers and charged collagen molecules, similar to eraser-based approaches on parchment^25^ and bone artifacts^9^. For the Pech I *lissoir*, we studied three separate samples. We used an available acetate surface replicate, initially non-destructively (similar to the membrane boxes) and subsequently by cutting off a small piece. Second, we sampled a plastic bag in which the Pech I *lissoir* had been stored for about five months. All attempts returned negative MALDI-TOF MS spectra of the Pech I *lissoir* (Table 3 and Supplementary Table S2).

The ammonium-bicarbonate buffer of each sample was processed following standard ZooMS collagen peptide mass fingerprinting^13^. In short, the buffer was heated to 65 °C for an hour to solubilize and denature the proteins recovered, cooled to 37 °C. Then, 1 μl of trypsin (Promega) was added for overnight digestion. Afterwards, the peptides were acidified using 10% TFA and cleaned on C18 ZipTips (Thermo Scientific). The peptides were analyzed on a MALDI-TOF MS at the IZI Fraunhofer, Leipzig, using previously published protocols^13^. Next, extracts were analyzed in mMass (v. 5.5.0)^77^ for the presence of collagen type I (COL1) peptide markers in comparison to a peptide marker database containing marker series for all medium- to larger-sized mammalian genera in existence in Europe during MIS5-3^13^.

Contamination of ancient proteins with modern proteins is a likely issue potentially affecting all steps involved in palaeoproteomics^78^. In addition, the use of a *lissoir* on animal skin, a protein-containing surface, might have transferred skin-specific proteins to the bone surface of the *lissoir*. Therefore, we employed a series of controls to check for the occurrence of protein contamination during excavation, storage, and laboratory analysis. These controls all followed the same extraction protocol detailed above. In addition, we employed *in silico* peptide matching to our MALDI-TOF MS fingerprints to determine the presence and/or absence of ancient, non-bone, proteins.

To begin, we performed two sets of blank extractions. The first was a normal laboratory blank alongside the *lissoir*-derived samples to check for the presence of protein in our reagents. The second was an extraction carried out on an empty membrane box that had not been used to store any archaeological or modern bone samples before. Both remained empty of collagenous peptides, demonstrating that the *lissoir* collagen fingerprints do not derive from modern laboratory or storage contamination (Fig. 2a).

Furthermore, we realized that bone specimens are often cleaned in water at field sites after recording to remove any adhering sediment. Theoretically, both the washing water and the sediment adhering to bone artifacts could contain proteins, creating opportunities for contamination. In order to test for this, we sampled washing water (*n* = 4) and archaeological sediment (*n* = 4) from an active excavation at Les Cottés, France. Les Cottés is a late Pleistocene archaeological site with demonstrated protein and ancient DNA survival^79–82^. Each sample was air dried in a 2 mL Eppendorf, resuspended in ammonium-bicarbonate, and subsequently processed identically to the Middle Paleolithic *lissoirs*. Just like the other extraction blanks, these also remained empty of any observable collagen peptides (Fig. 2b).

We also explored the possibility that protein might have been transferred from an animal skin to the bone artifact during use in the Pleistocene. In addition to skin-derived collagens, this likely would have included the transfer of skin keratins and common blood-derived proteins from herbivore species. We therefore generated an *in silico* peptide mass library of human COL1, keratin 1, and keratin 10, porcine trypsin, and *Bos* COL1 (CO1A1_BOVIN and CO1A2_BOVIN), keratin 1 (G3N0V2_BOVIN), keratin 10 (K1C10_BOVIN), BSA (ALBU_BOVIN), hemoglobin subunit alpha (HBA_BOVIN), and hemoglobin subunit beta (HBB_BOVIN). For each generated MALDI spectrum, monoisotopic peptide masses were picked in mMass with an S/N > 3. The resulting peak list was manually validated to remove any remaining isotope peaks not recognized due to deamidation. Subsequently, the reduced peak list was matched to the *in silico* peptide mass reference library with a maximum ppm error of 50. Protein identifications were only accepted with a minimum of two matching peptides in a spectrum. This resulted in accepted protein matches to *Bos* sp./*Bison* sp. COL1, porcine trypsin, and human keratins only. As no non-human skin-derived proteins were observed, we conclude that the endogenous component of the MALDI-TOF MS is exclusively composed of endogenous *Bos* sp./*Bison* sp. COL1.

Finally, we obtained a reference set of 40 bone specimens from Abri Peyrony (*n* = 24) and Pech I (*n* = 16), of which 38 were taxonomically identified based on morphological observation (note: two of these were misidentified). These bone specimens were all stored in plastic bags. For the Abri Peyrony specimens, we took the specimen out of the plastic bag and placed it in an unused membrane box. We then sampled the plastic bag. After leaving the bone specimen in the membrane box for at least 48 hours and opening this 15 – 20 times to generate friction between the bone and plastic surfaces, we also took a sample from the membrane box and a destructive sample from the bone specimen. For the Pech I samples, we took the specimen out of the plastic bag and sampled the bag only (no permission was available for destructive sampling). All protein extracts were performed as described above for the ammonium-bicarbonate buffer protocol, with identical taxonomic identifications as performed for the *lissoirs*. Additionally, the control bone specimens from Abri Peyrony were used to calculate the extent of deamidation of the peptide P1105 (sequence = GV*Q*GPPGPAGPR), for each of the three extraction methods separately^14,83^. Next, the same calculation was performed on the MALDI-TOF MS spectra obtained for the *lissoirs*.

### Zooarchaeological analysis

All piece-plotted fauna (>25 mm) and associated material from screening (6 – 25 mm) in Layers L-3A and L-3B at Abri Peyrony were analyzed. Whenever possible, each bone specimen was recorded to body size, species, skeletal element, side, and bone portion. Identifications were made using comparative collections at the University of California, Davis (Zooarchaeology Laboratory of the Department of Anthropology and Museum of Wildlife and Fish Biology) and the Musée National de Préhistoire, Les Eyzies-de-Tayac, France. The Pech I fauna was analyzed using similar methods for a previous study^52^. In order to maximize comparability across both zooarchaeological assemblages, only data ascribed to specific skeletal elements were included for further analyses^84^. This is the cause for any discrepancies in NISP data presented in Rendu^52^. Identifications based on size class were recorded differently between the two sites, so categories “small ungulate,” “medium ungulate,” and “large ungulate” were integrated with “ungulate size classes 1 and 1/2,” “ungulate size classes 2 and 2/3,” and “ungulate size class 3/4”, respectively. Here, we refer to taxonomic identifications based on size class by “small,” “medium,” and “large.” Because all *lissoirs* were produced on ribs, only the number of identified specimens (NISP) was calculated for this study. Given the difficulty in assigning ribs to a specific taxa and issues related to size variability within one animal, we chose not to calculate other metrics that are designed to assess the number of individuals in an assemblage.

### Statistical analysis

We used Bayesian reasoning to evaluate the relative support for competing hypotheses: *H*_0_: Neandertals in southwest France selected ribs for *lissoirs* opportunistically vs. *H*_*A*_: Neandertals in southwest France selected ribs of larger species for *lissoirs* strategically. The relative support for *H*_*A*_ vs. *H*_0_ is quantified by the posterior odds:

$$\frac{P(E\,{\rm{|}}\,{H}_{A})}{P(E\,{\rm{|}}\,{H}_{0})}\frac{P({H}_{A})}{P({H}_{0})}$$

where *E* represents a set of empirical observations (Supplementary Note). This form of reasoning appears in legal and forensic contexts where, for example, it must be decided whether crime scene evidence better supports the prosecution or defense^57,58^. *P*(*E* | *H*_0_) is the likelihood of the sample under H_0_, calculated using the multinomial formula with probabilities given by the relative frequencies of all rib fragments in an assemblage. Thus under H_0_, Neandertals randomly selected ribs for making *lissoirs* in proportion to the prey species in each assemblage. *P*(*E* | *H*_*A*_) is the likelihood of the sample under H_A_, calculated using the Dirichlet-multinomial distribution^85^, which incorporates selectivity values derived from the natural logarithms of animal masses (Supplementary Table S6) and represents hypothesized functional preferences for ribs from larger-bodied, more robust taxa. Stronger and weaker versions of H_A_ are considered by rescaling the selectivity values (Supplementary Note). The prior odds *P*(*H*_*A*_)/*P*(*H*_0_), expressing the state of knowledge about the hypotheses before evidence is collected, are required to complete the calculation. As both the prior and posterior odds are merely statements about *relative* support, the two hypotheses need not be exhaustive or mutually exclusive. Given our limited knowledge of Neandertal procurement and manufacture of bone tools, we have no *a priori* preference for one hypothesis over the other, therefore we use a prior odds of 1. Prior odds can be adjusted based on subsequent findings.

## Supplementary information

Supplementary Information

## Data Availability

All data generated or analyzed during this study are included in this published article (and its Supplementary Information files).
