# Supplementary material for: Non-destructive ZooMS identification reveals strategic bone tool raw material selection by Neandertals
Source: Sci Rep. 2020 May 8;10:7746. doi: 10.1038/s41598-020-64358-w (PMC7210944; doi:10.1038/s41598-020-64358-w)
Supplement: Supplementary file 1 — Supplementary Information [file 41598_2020_64358_MOESM1_ESM.pdf]

Supplementary Information for **Non-destructive ZooMS identification reveals strategic bone tool raw material selection by Neandertals**

Naomi L. Martisius, Frido Welker, Tamara Dogandžić, Mark N. Grote, William Rendu, Virginie Sinet-Mathiot, Arndt Wilcke, Shannon J.P. McPherron, Marie Soressi, and Teresa E. Steele

Table of Contents

Supplementary Figures……………………………………..……………..…………….….2

Supplementary Fig. S1. Destructive ZooMS sampling on one of the reference fauna specimens from Abri Peyrony (AP-4957)…………………….……………………………..2

Supplementary Fig. S2. Animal size class composition of all skeletal elements compared to ribs by NISP of the layers that preserved the *lissoirs* at Pech I and Abri Peyrony……..…3

Supplementary Fig. S3. Membrane box used for curating one of the Abri Peyrony *lissoirs* (AP-7839) for more than five years………………………….……………….……..…4

Supplementary Fig. S4. Deamidation (peptide P1105) observed in MALDI-TOF MS spectra obtained through destructive or non-destructive approaches……………………………...5

Supplementary Fig. S5. Possible Middle Paleolithic *lissoir* from La Quina….…………………6

Supplementary Tables…………………………………………………….…….……….……7

Supplementary Table S1. *Lissoir* dimensions and rib completeness…………….……….…..…7

Supplementary Table S2. Peptide marker masses observed in the five *lissoirs* studied…..……7

Supplementary Table S3. List of taxa found in the Dordogne associated with Post-Quina technologies ……………………………………………………….…………….………..8

Supplementary Table S4. Results of ZooMS analysis on reference fauna specimens from Abri Peyrony and Pech-de-l'Azé I……………………………………………....……9-10

Supplementary Table S5. Likelihoods of the null and alternative hypotheses and the posterior odds of the alternative hypotheses given the null for the separate layers at Abri Peyrony …………….…………………………………………...…...…………….…...10

Supplementary Table S6. List of animals used in the Dirichlet-multinomial model as possible raw material sources for *lissoirs*……………………….…………………….……11

Supplementary Notes…………………………………..………………………………. 12

Relative support for competing hypotheses about *lissoir* selection……..…….…………..…… 12

Statistical methodology……………………...……………………………………...………13

Results…………………………….……………….……………………………...……16

Supplementary Information References…….……………………….…….……….…...…..…18

Supplementary Figures

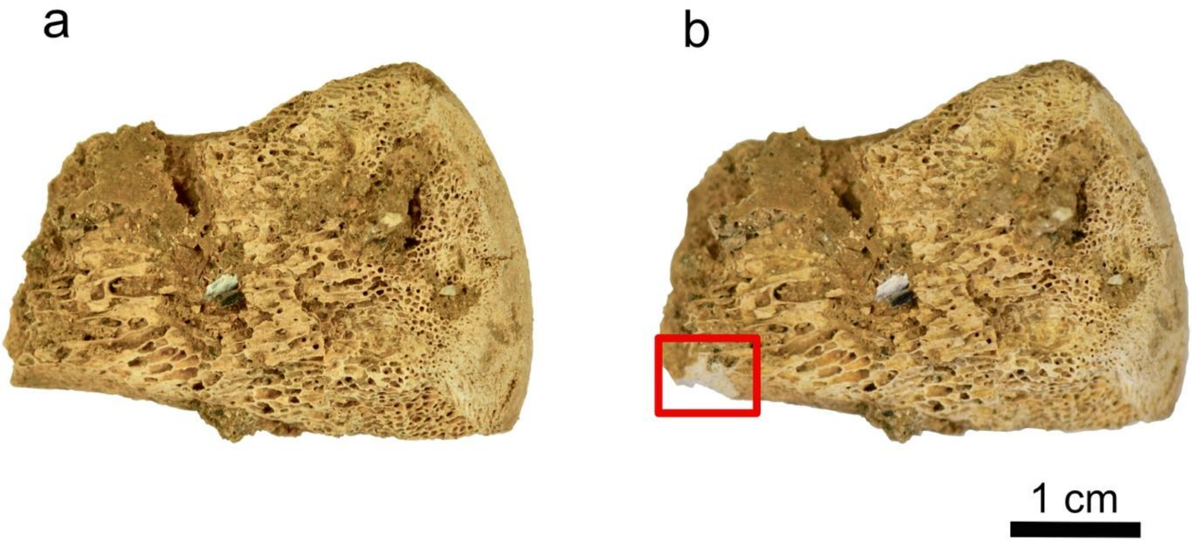

Supplementary Fig. S1. Destructive ZooMS sampling on one of the reference fauna specimens from Abri Peyrony (AP-4957). (**a**) Before sampling. (**b**) After sampling. The red box highlights where the specimen was sampled.

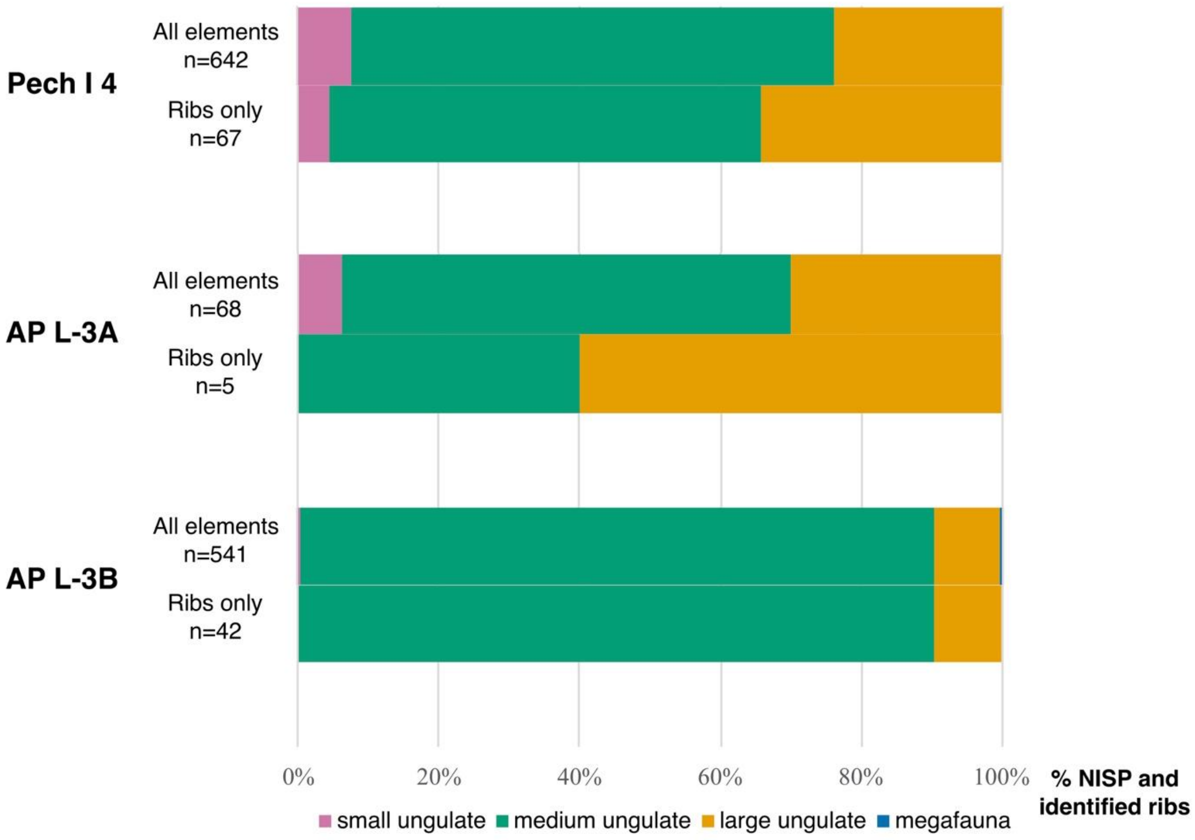

Supplementary Fig. S2. Animal size class composition of all skeletal elements compared to ribs by NISP of the layers that preserved the *lissoirs* at Pech I and Abri Peyrony (AP). Within each assemblage, the upper bar is the percent animal size class of identified specimens, and the lower bar is the percent animal size class of ribs.

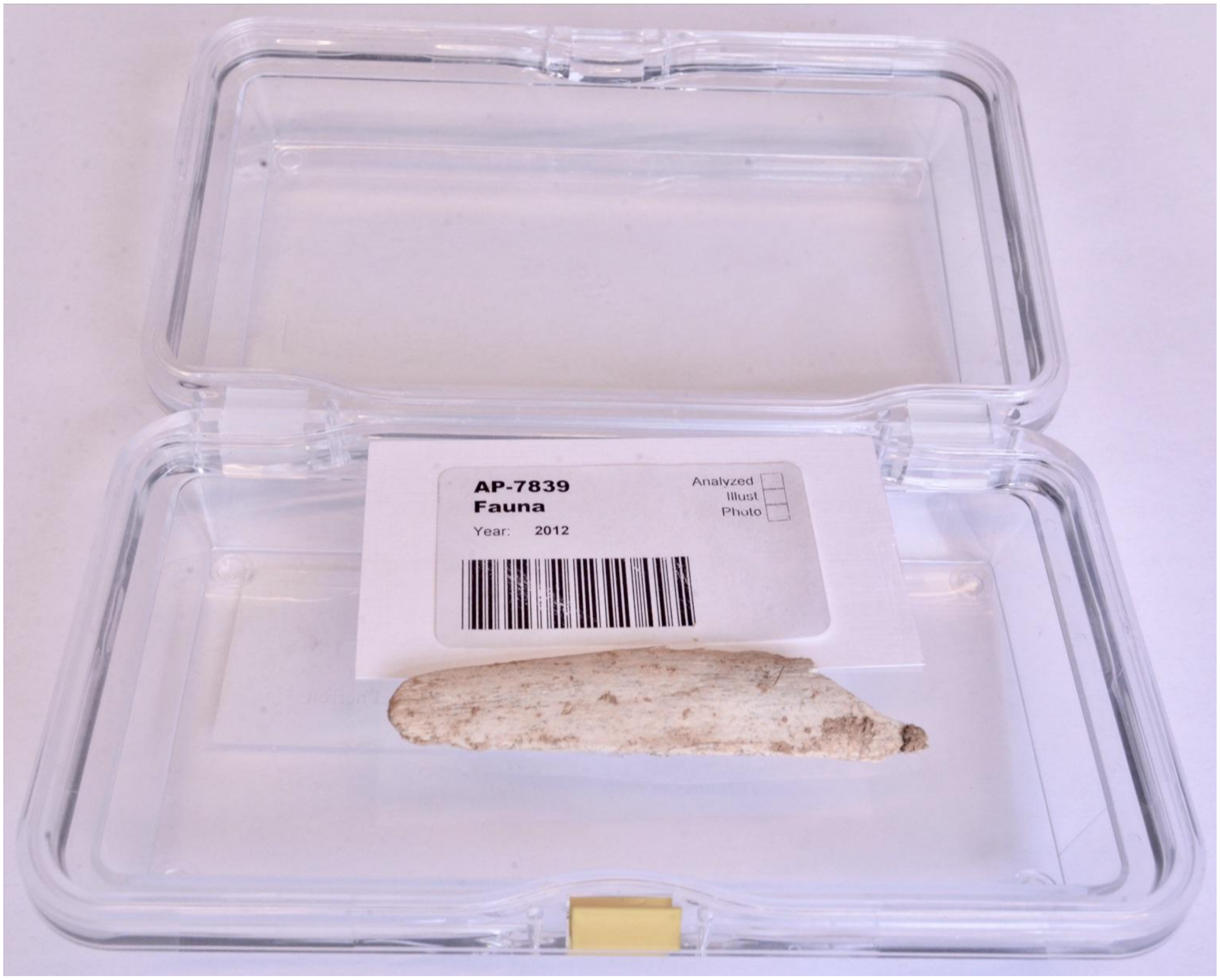

Supplementary Fig. S3. Membrane box used for curating one of the Abri Peyrony *lissoirs* (AP-7839) for more than five years.

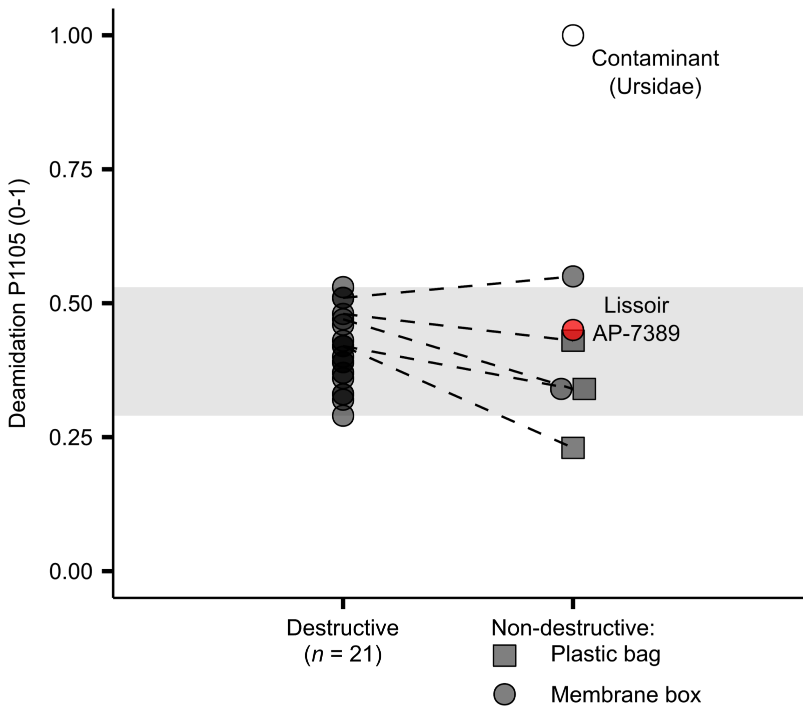

Supplementary Fig. S4. Deamidation (peptide P1105) observed in MALDI-TOF MS spectra obtained through destructive or non-destructive approaches. The range of deamidation observed in the destructive extractions is shaded. Bone specimens for which deamidation values were available using both destructive and non-destructive extraction methods are joined by dashed lines. Deamidation could be calculated reliably for just one *lissoir* (in red). Deamidation ranges from 1.0, no deamidation observed for any glutamines, to 0.0, complete deamidation of all glutamines. The protein present in the membrane extraction of AP-3597, identified as "Ursidae" likely represents a contaminant, as also supported by its deamidation value (1.0, also see the main text).

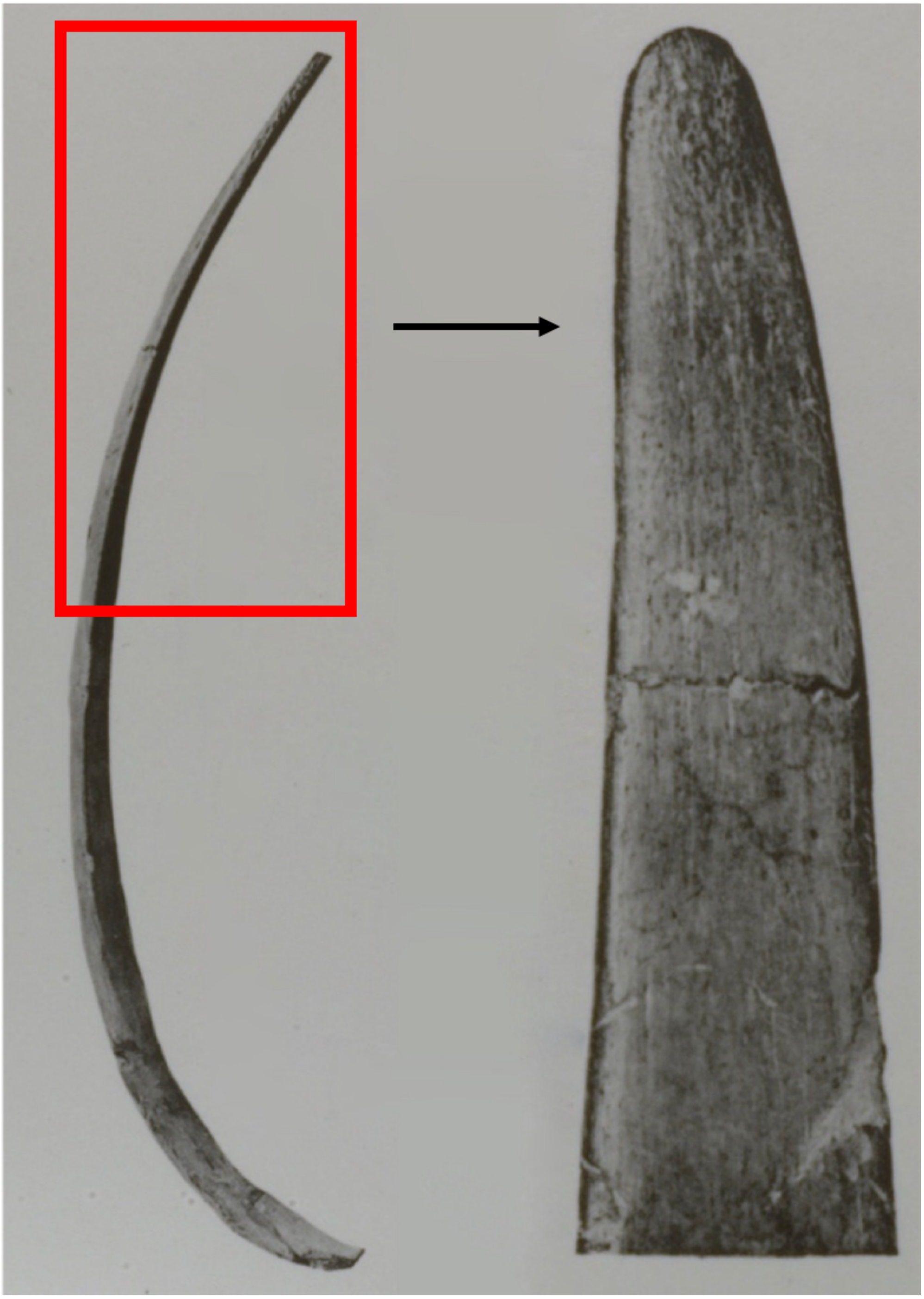

Supplementary Fig. S5. Possible Middle Paleolithic *lissoir* from La Quina. Images after Martin[1], pl. XXV.

Supplementary Tables

Specimen ID number	Max length [*mm*]	Max width [*mm*]	Max thickness [*mm*]	Half or complete rib
PA I G8-1417	33.3	16.1	2.9	Half
AP-4209	21.1	14.8	4.8	Half
AP-4493	20.9	12.7	3.8	Half
AP-7839	82.7	20.1	5.9	Complete
AP-10818	14.7	11.5	1.9	Half

Supplementary Table S1. *Lissoir* dimensions and rib completeness. The complete *lissoir* preserves the double layer of cortical bone, while the half *lissoirs* preserve one layer.

Sample	Marker P1	Marker A	Marker B	Marker C	Marker P2	Marker D	Marker E	Marker F	Marker G	Taxonomic ID
PA I G8-1417	x	x	1427	x	x	x	x	x	x	Unidentifiable
AP-4209	1105	1208	1427	1580	1648	2131	x	2853	x	*Bison* sp./*Bos* sp.
AP-4493	1105	1208	1427	1580	1648	2131	2792	2869	x	*Bison* sp./*Bos* sp.
AP-7839	1105	1208	1427	1580	1648	2131	x	x	x	*Bison* sp./*Bos* sp.
AP-10818	1105	1208	1427	1580	1648	2131	x	x	x	*Bison* sp./*Bos* sp.

Supplementary Table S2. Peptide marker masses observed in the five *lissoirs* studied. Marker names based on[2,3].

Site	Post-Quina Levels	All Taxa Reported	References
**Abri Peyrony**	L-3A, L-3B, U-3	rabbit, roe deer, red deer, reindeer, large bovid, woolly rhinoceros, medium carnivore, and large carnivore	Soressi et al. 2013; Martisius et al. 2015
**Chapelle aux Saints - Bouffia 118**	alph, c2	fox, hyena, wild cat, reindeer, roe deer, Cervid, bovid, horse, avifauna, and marmot	Rendu et al. 2014
**Combe-Grenal**	Layer 11-16	large bovid, ibex, horse, red deer, chamois, Europena ass, reindeer, hyena, fox and wolf	Discamps and Faivre 2017; Guadelli 1987; Laquay 1981; Marquet 1993
**La Ferrassie**	5 to 3	bison, bovid, red deer, reindeer, horse, roe deer, woolly rhinoceros, woolly mammoth (in burrow, per comm.), bear, hyena, wild boar, fox, and wolf	Guérin et al. 2015
**Le Moustier**	H4-H9, H2, G1-G2	large bovid, chamois, horse, ibex, red deer, and reindeer	Valladas et al. 1986; Gravina and Discamps 2015
**Pech-de-l'Azé I**	7 to 4	fox, rabbit, roe deer, red deer, reindeer, bison, horse, and avifauna	Marquet 1993; Soressi et al. 2007, 2008, 2013; Rendu 2010
**Pech-de-l'Azé IV**	3	roe deer, red deer, reindeer and large bovid	Laquay 1981; Dibble et al. 2009; McPherron et al. 2012; Niven 2013; Richter et al. 2013

Supplementary Table S3: List of taxa found in the Dordogne associated with Post-Quina technologies. Adapted from[4] and compiled from[5-21].

Site	Squid	Morphological identification	Destructive ZooMS	Plastic bag-based ZooMS	Membrane box-based ZooMS
**Abri Peyrony**	AP-750	**Unidentifiable**	Carnivora	Empty	Empty
	AP-3229	**Unidentifiable**	*Bison* sp./*Bos* sp.	Empty	Empty
	AP-4183	*Bison* sp./*Bos* sp.*	Cervid/Saiga	Bovidae/Cervidae	Empty
	AP-423	*Bison* sp./*Bos* sp.	*Bison* sp./*Bos* sp.	Empty	Empty
	AP-8696	*Bison* sp./*Bos* sp.	*Bison* sp./*Bos* sp.	*Bison* sp./*Bos* sp.	Empty
	AP-6953	*Bison* sp./*Bos* sp.	*Bison* sp./*Bos* sp.	Bovidae/Cervidae	Empty
	AP-4514	*Bison* sp./*Bos* sp.	*Bison* sp./*Bos* sp.	Empty	**Unidentifiable**
	AP-4957	*Bison* sp./*Bos* sp.	*Bison* sp./*Bos* sp.	Empty	Empty
	AP-573	*Bison* sp./*Bos* sp.	*Bison* sp./*Bos* sp.	Empty	Empty
	AP-514	*Bison* sp./*Bos* sp.	*Bison* sp./*Bos* sp.	*Bison* sp./*Bos* sp.	Empty
	AP-2106	*Bison* sp./*Bos* sp.	*Bison* sp./*Bos* sp.	**Unidentifiable**	Empty
	AP-5863	*Bison* sp./*Bos* sp.	*Bison* sp./*Bos* sp.	*Bison* sp./*Bos* sp.	*Bison* sp./*Bos* sp.
	AP-5661	*Bison* sp./*Bos* sp.*	*Rangifer tarandus*	Bovidae/Cervidae	Empty
	AP-7049	*Rangifer tarandus*	*Rangifer tarandus*	*Rangifer tarandus*	*Rangifer tarandus*
	AP-7151	*Rangifer tarandus*	*Rangifer tarandus*	Empty	**Unidentifiable**
	AP-3597	*Rangifer tarandus*	Empty	Ursidae*	Empty
	AP-2541	*Rangifer tarandus*	*Rangifer tarandus*	Empty	**Unidentifiable**
	AP-2567	*Rangifer tarandus*	*Rangifer tarandus*	Empty	Empty
	AP-5246	*Rangifer tarandus*	*Rangifer tarandus*	Empty	Empty
	AP-7048.1	*Rangifer tarandus*	*Rangifer tarandus*	Empty	Empty
	AP-2050.3	*Rangifer tarandus*	*Rangifer tarandus*	Empty	Empty
	AP-5156.1	*Rangifer tarandus*	*Rangifer tarandus*	Empty	Empty
	AP-8338	*Rangifer tarandus*	*Rangifer tarandus*	*Rangifer tarandus*	Empty
	AP-6705	*Rangifer tarandus*	**Unidentifiable**	Empty	Empty
**Pech-de-l'Azé I**	G8-1444	*Bison* sp./*Bos* sp.	Not tested.	*Bison* sp./*Bos* sp.	Not tested.
	G8-211	*Bison* sp./*Bos* sp.		*Bison* sp./*Bos* sp.	
	G8-621	*Bison* sp./*Bos* sp.		Empty	
	G9-945	*Bison* sp./*Bos* sp.		Empty	
	H8-82	*Bison* sp./*Bos* sp.		Empty	
	K14-53	*Bison* sp./*Bos* sp.		**Unidentifiable**	
	H9-18	*Bison* sp./*Bos* sp.		*Bison* sp./*Bos* sp.	
	H9-25	*Bison* sp./*Bos* sp.		Empty	
	G8-704	*Cervus*		Cervid/Saiga/Roe	
	H8-121	*Cervus*		Empty	
	H8-81	*Cervus*		Empty	
	H8-91	*Cervus*		Empty	
	H9-38	*Cervus*		Empty	
	J15-132	*Cervus*		Empty	
	G8-545	*Cervus*		Empty	
	H8-201	*Cervus*		Empty	

Supplementary Table S4: Results of ZooMS analysis on reference fauna specimens from Abri Peyrony and Pech-de-l'Azé I. Abri Peyrony specimens were sampled destructively and non-destructively in two different ways. Pech-de-l'Azé I specimens were sampled using one non-destructive method. Non-destructive identifications are generally in agreement with the morphological and destructive ZooMS identities. Three identifications indicated with * are inconsistent with the other results for that specimen. Two specimens (AP-4183 and AP-5661) morphologically misidentified as *Bison* sp./*Bos* sp. consistently provided ZooMS results of differing taxa. The plastic bag identified as Ursidae (AP-3597) is inconsistent with the destructive extraction and the morphological identification and is likely the result of contamination prior to laboratory analysis. A deamidation value of 1.0 supports this interpretation (see Supplementary Fig. S4).

	**L-3A**		**L-3B**	
	***P*(*E* | *H*)**	**Posterior Odds H_A:H_0**	***P*(*E* | *H*)**	**Posterior Odds H_A:H_0**
**Null (H_0)**	0.6	—	0.0009	—
**Alternative strong (H_{As})**	0.125	0.2	0.0028	3.2
**Alternative weak (H_{Aw})**	0.125	0.2	0.0037	4.2

Supplementary Table S5: Likelihoods of the null and alternative hypotheses and the posterior odds of the two hypotheses for the separate layers at Abri Peyrony. Likelihoods were calculated using combined counts of rib fragments of *Bison* sp./*Bos* sp. and large ungulates as shown in Table 2 of the main text, divided by the total number of rib fragments if appropriate (as for H_0). Numerical values above have been rounded.

Taxon	Latin name	Weight [*kg*]	Natural log of mid-range weight (w)	Supplementary information
Rhinoceros	Rhinocerotidae	1,400-3,600	7.8	White rhinoceros used as modern analog
Aurochs and Bison	Bovinae	350-1000	6.5	
Horse	*Equus caballus*	200-300	5.5	Przewalski's horse used as modern analog
Red deer	*Cervus elaphus*	75-340	5.3	
European ass	*Equus hydruntinus*	180-200	5.2	Chabai et al 2007
Reindeer	*Rangifer tarandus*	60-318	5.2	
Wild boar	*Sus scrofa*	60-270	5.1	Tack 2018
Ibex	*Capra* sp.	35-150	4.5	
Chamois	*Rupicapra rupicapra*	24-50	3.6	
Roe deer	*Capreolus capreolus*	15-50	3.5	

Supplementary Table S6: List of animals used in the Dirichlet-multinomial model as possible raw material sources for *lissoirs*. Animal mass ranges taken from[22], except where specified[23,24]. The selectivity values for the strong alternative hypothesis are given as the natural logarithm of each taxon's mid-range weight.

Supplementary Notes

Relative support for competing hypotheses about *lissoir* selection

The ability to discriminate species through the application of ZooMS permits the consideration of competing hypotheses about selection of ribs used for *lissoirs*. Availability, suitability of skeletal element shape, and quality of the bone element and/or species[25] are important characteristics when selecting a raw material source to make bone tools. Whether Neandertals took these factors into account when selecting, making, and using bone tools is a matter that needs further consideration. It stands to reason that Neandertals derived tools from bones of the animals that they hunted for subsistence purposes, but bones used as tools may have been chosen opportunistically from the available lot. For instance, bone retouchers—bone fragments opportunistically utilized to shape flint artifacts—have been shown to originate from commonly hunted species, and their taxonomic frequencies are similar to the overall distribution of taxa in faunal assemblages[26-29 but see 30]. It is possible that Neandertals showed a similar lack of selectivity when utilizing ribs—the raw material source for making *lissoirs*. Alternatively, Neandertals used the same skeletal element (rib) for making *lissoirs*, which provides a line of evidence for Neandertal selectivity. In addition, the highly standardized final shape and size of the five Pech I and Abri Peyrony *lissoirs* could imply that Neandertals selected the ribs from species of a certain size. Thicknesses of the *lissoirs* (Supplementary Table S1) could be consistent with rib dimensions of a larger animal than initially considered. During our previous study[16], using standard morphological assessments, we determined that four *lissoirs* were produced on ribs of medium-sized ungulates. Because the bones are highly fragmented and

modified, species determinations were challenging. Ribs from a larger animal should provide a stronger implement during use. If Neandertals consistently selected the ribs of the larger animals in their environment, this could provide evidence for strategic raw material selection. Given this, applying non-destructive ZooMS to the five Middle Paleolithic *lissoirs* provides an opportunity to test competing propositions about Neandertal selection of ribs for functional purposes.

Consider the following hypotheses:

H_0: Neandertals in southwest France selected ribs for *lissoirs* opportunistically

H_A: Neandertals in southwest France selected ribs of larger species for *lissoirs* strategically.

Statistical methodology

We used Bayesian reasoning to evaluate the relative support for competing hypotheses in light of material evidence E, a set of empirical observations. This form of reasoning appears in legal and forensic contexts where, for example, it must be decided whether crime scene evidence better supports the prosecution or defense[31,32].

Under the null hypothesis (H_0), we predict that Neandertals randomly selected ribs for making *lissoirs* in proportion to the prey species in each assemblage. Here, we calculate the likelihood of the sample under H_0 using the multinomial formula with probabilities given by the relative frequencies of all rib elements in an assemblage:

$$P(E\,|\,H_0) = [n!/(x_1!x_2!\ldots x_k!)](p_1^{x_1}p_2^{x_2}\ldots p_k^{x_k}),$$

where n is the total number of *lissoirs* in the assemblage; $x_1, x_2, \ldots x_k$ are the *lissoir* counts from taxa 1, 2, ... k, with $\Sigma_i x_i = n$; and $p_1, p_2, \ldots p_k$ are the frequencies in the assemblage of rib elements of taxa 1, 2, ... k. Given that all *lissoirs* at Abri Peyrony were made from large bovid ribs, the

multinomial equation simplifies to $P(E \mid H_0) = p_{bb}{}^{x_{bb}}$, where "*bb*" indicates the taxon *Bos* sp./*Bison* sp.

Under the alternative hypothesis (H_A), we predict that Neandertals strategically selected ribs from larger taxa for making *lissoirs*. Here, we incorporate hypothesized functional preferences by taxon, and calculate the likelihood of the sample under H_A using the Dirichlet-multinomial distribution[33]:

$$P(E \mid H_A) = nB(\Sigma_i w_i, n)/[\prod_{i:x_i>0} x_i B(w_i, x_i)],$$

where B() is the Beta function and w_1, w_2, … w_k are non-negative parameters of the Dirichlet distribution reflecting selectivity values of taxa 1, 2, ... k, described below. Given that all *lissoirs* at Abri Peyrony were made from large bovid ribs, the Dirichlet-multinomial equation simplifies to $P(E \mid H_A) = B(\Sigma_i w_i, x_{bb})/B(w_{bb}, x_{bb})$.

Following Discamp et al.[4], we compile a list of animals found at archaeological sites in the Dordogne, the region in France where Abri Peyrony and Pech I are located, and from the time period associated with the assemblages (Supplementary Table S3). We exclude carnivores and animals smaller than roe deer as possible raw material sources, because the shape and size of these ribs are arguably inappropriate for manufacturing *lissoirs*. We also exclude woolly mammoth, because the only example from the compiled list (Supplementary Table S3, La Ferrassie) was found in an animal burrow making its provenience unclear (TES and SPM, personal observation). The list of taxa used in the model (woolly rhinoceros, large bovids, horse, red deer, European ass, reindeer, wild boar, ibex, chamois, and roe deer) reflects all observed ungulates in the region during late Neandertal time periods and does not distinguish whether an animal was abundant or sparse on the landscape. Given that the alternative hypothesis predicts

that larger ribs are preferred for making *lissoirs*, the Dirichlet-multinomial distribution integrates this "selectivity" using different values w_1, w_2, … w_k representing hypothesized preferences for each taxon's rib. The more preferred animal ribs (i.e., larger ribs) are assigned relatively larger selectivity values, while the less preferred (i.e., smaller ribs) are assigned smaller values. Logically, larger ribs are attributed to larger taxa, and because there is a power-law relationship between animal mass and ribcage size (see e.g.,[34-36]), the selectivity values (w) are derived from the natural logarithms of animal masses (Supplementary Table S6).

The selectivity values w_1, w_2, … w_k in the Dirichlet-multinomial equation can be used to incorporate prior scientific knowledge, as well as rational argument, and they allow for considerable flexibility in specifying the strength of the alternative hypothesis. In particular, stronger (H_{As}) and weaker (H_{Aw}) forms of the alternative are obtained by rescaling the entire set of selectivity values: a large-magnitude set w_1, w_2, … w_k implies a stronger prior belief that the frequencies of rib elements used for *lissoirs* will closely hew to the selectivity values, whereas a small-magnitude set w_1, w_2, … w_k implies more prior uncertainty. This uncertainty is reflected in a larger standard deviation for the hypothetical number of *lissoirs* made from each taxon under the alternative. For the strong alternative hypothesis we use the natural logarithms of animal masses as selectivity values [$w_i = log(\text{mass}_i)$]. Although larger-bodied animals are certainly preferred under the strong alternative, the power-law relationship (see e.g.,[34-36]) implies diminishing returns to the utility of ribs as animal mass increases. For the weak alternative hypothesis we rescale the selectivity values by dividing by two [$w_i' = log(\text{mass}_i)/2$], implying more severely diminishing returns, and at the same time more uncertainty about the expected number of *lissoirs* per species under the alternative.

The relative support for H_0 vs. H_A is quantified by the posterior odds[31,32]:

$$\frac{P(E \mid H_A)}{P(E \mid H_0)} \frac{P(H_A)}{P(H_0)},$$

where the first factor $P(E \mid H_A)/P(E \mid H_0)$ is known as the likelihood ratio and the second factor $P(H_A)/P(H_0)$ is the prior odds. The prior odds $P(H_A)/P(H_0)$, expressing the state of knowledge about the hypotheses before evidence is collected, are required to complete the calculation. As both the prior and posterior odds are merely statements about *relative* support, the two hypotheses need not be exhaustive or even mutually exclusive. Given our limited knowledge of Neandertal procurement and manufacture of bone tools, we have no *a priori* preference for one hypothesis over the other, therefore we use a prior odds of 1. Prior odds can be adjusted based on subsequent findings.

Results

Likelihoods and posterior odds are displayed in Supplementary Table S5 and discussed in the section *Relative support for competing hypotheses* of the main text. Briefly, the null hypothesis of opportunistic selection is better supported in Layer L-3A, but the alternative hypothesis of strategic selection of large-bodied ungulate ribs, in either the strong or weak form, is better supported in Layer L-3B.

Though both alternative models are supported in Layer L-3B, an interpretation of their differential support, in light of Neandertals' likely encounter rates with prey taxa, can be entertained. Inherent in the strong alternative model is the assumption that rib selection according to logarithmic animal body mass would be rather strictly followed. For example, because woolly rhinoceros has the largest ribs, their ribs should be used most often, constituting

7.8/52.2, or about 15%, of *lissoirs* on average. This relative frequency is the selectivity value *w* for woolly rhinoceros (Supplementary Table S6), divided by the sum of all selectivity values. Both the strong and weak alternatives imply the same relative frequency on average. However, the weak alternative allows for more variance in relative frequencies across assemblages[33]. The strong alternative model, in particular, is indifferent to the possibility that woolly rhinoceros may have been rare on the Neandertal landscape, and therefore less likely to be used. It is probable that Neandertals would select the ribs from the animals they were already hunting, and zooarchaeological evidence shows that Neandertals rarely exploited woolly rhinoceros, especially in the region and time period considered here (Supplementary Table S3). Furthermore, at the two sites (La Ferrassie and Abri Peyrony) where woolly rhinoceros is present, they are only represented as an NISP of 1[19,20]. The Neandertals in this time simply may not have used woolly rhinoceros ribs to make *lissoirs*, because their ribs were not frequently available to them. Instead, they chose the ribs of the largest animals more abundant on the landscape to make *lissoirs*. The weak alternative hypothesis is more accommodating of this possibility.

Supplementary Information References

1 Martin, H. Recherches sur l'Evolution du Moustérien dans le Gisement de La Quina (Charente) Vol.1, Fasc. 2. Ossements Utilisés. (1909).

2 Buckley, M., Collins, M., Thomas-Oates, J. & Wilson, J. C. Species identification by analysis of bone collagen using matrix-assisted laser desorption/ionisation time-of-flight mass spectrometry. *Rapid Commun Mass Spectrom* **23**, 3843-3854, doi:10.1002/rcm.4316 (2009).

3 Kirby, D. P., Buckley, M., Promise, E., Trauger, S. A. & Holdcraft, T. R. Identification of collagen-based materials in cultural heritage. *Analyst* **138**, 4849-4858, doi:10.1039/c3an00925d (2013).

4 Discamps, E. & Royer, A. Reconstructing palaeoenvironmental conditions faced by Mousterian hunters during MIS 5 to 3 in southwestern France: A multi-scale approach using data from large and small mammal communities. *Quaternary International* **433**, 64-87, doi:10.1016/j.quaint.2016.02.067 (2017).

5 Laquay, G. *Recherches sur les faunes du Würm I en Périgord* PhD dissertation thesis, Université Bordeaux 1, (1981).

6 Valladas, H., Geneste, J., Joron, J. & Chadelle, J. Thermoluminescence dating of Le Moustier (Dordogne, France). *Nature* **322**, 452 (1986).

7 Guadelli, J.-L. Contribution à l'étude des zoocénoses préhistoriques en Aquitaine: Würm ancien et interstade würmiem PhD dissertation thesis, Université de Bordeaux I, (1987).

8 Marquet, J.-C. in Les Cahiers de la Claise (1993).

9 Soressi, M., Jones, H. L., Rink, W. J., Maureille, B. & Tillier, A. M. The Pech-de-l'Aze I Neandertal child: ESR, uranium-series, and AMS 14C dating of its MTA type B context. *J Hum Evol* **52**, 455-466, doi:10.1016/j.jhevol.2006.11.006 (2007).

10 Soressi, M. et al. in Les sociétés Paléolithiques d'un grand Sud-Ouest: nouveaux gisements, nouvelles méthodes, nouveaux résultats (eds J Jaubert, J-G Bordes, & I Ortega) 95-132 (Société Préhistorique française, 2008).

11 Dibble, H. L. *et al.* A preliminary report on Pech de l'Azé IV, layer 8 (Middle Paleolithic, France). *PaleoAnthropology* **2009**, 182-219 (2009).

12 Rendu, W. Hunting behavior and Neanderthal adaptability in the Late Pleistocene site of Pech-de-l'Aze I. *Journal of Archaeological Science* **37**, 1798-1810, doi:10.1016/j.jas.2010.01.037 (2010).

13 McPherron, S. P. *et al.* Radiocarbon dates for the late Middle Palaeolithic at Pech de l'Aze IV, France. *Journal of Archaeological Science* **39**, 3436-3442, doi:10.1016/j.jas.2012.05.017 (2012).

14 Niven, L. in Zooarchaeology and Modern Human Origins 151-161 (Springer, 2013).

15 Richter, D. *et al.* The late Middle Palaeolithic in Southwest France: New TL dates for the sequence of Pech de l'Aze IV. *Quaternary International* **294**, 160-167, doi:10.1016/j.quaint.2012.05.028 (2013).

16 Soressi, M. *et al.* Neandertals made the first specialized bone tools in Europe. *Proc Natl Acad Sci U S A* **110**, 14186-14190, doi:10.1073/pnas.1302730110 (2013).

17 Rendu, W. *et al.* Evidence supporting an intentional Neandertal burial at La Chapelle-aux-Saints. *Proceedings of the National Academy of Sciences of the United States of America* **111**, 81-86, doi:10.1073/pnas.1316780110 (2014).

18 Gravina, B. & Discamps, E. MTA-B or not to be? Recycled bifaces and shifting hunting strategies at Le Moustier and their implication for the late Middle Palaeolithic in southwestern France. *Journal of human evolution* **84**, 83-98 (2015).

19 Guérin, G. *et al.* A multi-method luminescence dating of the Palaeolithic sequence of La Ferrassie based on new excavations adjacent to the La Ferrassie 1 and 2 skeletons. *Journal of Archaeological Science* **58**, 147-166 (2015).

20 Martisius, N. L., Dogand žić, T., Lenoir, M., McPherron, S. P. & Steele, T. E. in *Society for American Archaeology 80th Annual Meeting* (San Francisco, California, 2015).

21 Discamps, E. & Faivre, J. P. Substantial biases affecting Combe-Grenal faunal record cast doubts on previous models of Neanderthal subsistence and environmental context. *Journal of Archaeological Science* **81**, 128-132, doi:10.1016/j.jas.2017.03.009 (2017).

22 Nowak, R. M. *Walker's Mammals of the World*. 5 edn, Vol. I & II (Johns Hopkins University Press, 1991).

23 Chabai, V., Richter, J. & Uthmeier, T. Palaeolithic Sites of Crimea 3.2: KABAZI V: Interstratification Of Micoquian & Levallois-Mousterian Camp Sites. Vol. 3 (University of Cologne, 2007).

24 Tack, J. Wild boar (Sus scrofa) populations in Europe: a scientific review of population trends and implications for management. 56 (European Landowners' Organization, Brussels, 2018).

25 Choyke, A. M. in From these bare bones: raw materials and the study of worked osseous objects (eds Alice Choyke & Sonia O'Connor) Ch. 1, 1-11 (Oxbow Books, 2013).

26 Costamagno, S. *et al.* in *The Origins of Bone Tool Technologies* (eds Jarod M. Hutson & Elisabeth S. Noack) 165-196 (Römisch-Germanischen Zentralmuseums, 2018).

27 Daujeard, C. *et al.* in *The Origins of Bone Tool Technologies* (eds Jarod M. Hutson & Elisabeth S. Noack) 93-132 (Römisch-Germanischen Zentralmuseums, 2018).

28 Sévêque, N. & Auguste, P. in *The Origins of Bone Tool Technologies* (eds Jarod M. Hutson & Elisabeth S. Noack) 133-164 (Römisch-Germanischen Zentralmuseums, 2018).

29 Thun Hohenstein, U., Bertolini, M., Channarayapatna, S., Modolo, M. & Peretto, C. in *The Origins of Bone Tool Technologies* (eds Jarod M. Hutson & Elisabeth S. Noack) 235-250 (Römisch-Germanischen Zentralmuseums, 2018).

30 Rougier, H. *et al.* Neandertal cannibalism and Neandertal bones used as tools in Northern Europe. *Sci Rep* **6**, 29005, doi:10.1038/srep29005 (2016).

31 Evett, I. The logical foundations of forensic science: towards reliable knowledge. *Philos Trans R Soc Lond B Biol Sci* **370**, 20140263, doi:10.1098/rstb.2014.0263 (2015).

32 Fenton, N., Neil, M. & Berger, D. Bayes and the Law. *Annu Rev Stat Appl* **3**, 51-77, doi: 10.1146/annurev-statistics-041715-033428 (2016).

33 Johnson, N. L., Kotz, S. & Balakrishnan, N. *Discrete multivariate distributions*. Vol. 165 (John Wiley & Sons, Inc., 1997).

34 McEwan, E. & Wood, A. Growth and development of the barren ground caribou: I. heart girth, hind foot length, and body weight relationships. *Canadian Journal of Zoology* **44**, 401-411 (1966).

35 Cook, R. C., Cook, J. G. & Irwin, L. L. Estimating elk body mass using chest-girth circumference. *Wildlife Society Bulletin* **31**, 536-543 (2003).

36 Anzai, H. *et al.* Interspecific comparison of allometry between body weight and chest girth in domestic bovids. *Scientific Reports* **7**, 4817, doi:ARTN 4817 10.1038/s41598-017-04976-z (2017).
